# Understanding Barriers to Participation in Cost-Share Programs For Pollinator Conservation by Wisconsin (USA) Cranberry Growers

**DOI:** 10.3390/insects8030079

**Published:** 2017-08-01

**Authors:** Hannah R. Gaines-Day, Claudio Gratton

**Affiliations:** Department of Entomology, University of Wisconsin-Madison, Madison, WI 53706, USA; cgratton@wisc.edu

**Keywords:** bees, agri-environment scheme, pollinator habitat, two-wave mail survey, program participation, EQIP, CRP, classification tree analysis, USDA Farm Bill

## Abstract

The expansion of modern agriculture has led to the loss and fragmentation of natural habitat, resulting in a global decline in biodiversity, including bees. In many countries, farmers can participate in cost-share programs to create natural habitat on their farms for the conservation of beneficial insects, such as bees. Despite their dependence on bee pollinators and the demonstrated commitment to environmental stewardship, participation in such programs by Wisconsin cranberry growers has been low. The objective of this study was to understand the barriers that prevent participation by Wisconsin cranberry growers in cost-share programs for on-farm conservation of native bees. We conducted a survey of cranberry growers (n = 250) regarding farming practices, pollinators, and conservation. Although only 10% of growers were aware of federal pollinator cost-share programs, one third of them were managing habitat for pollinators without federal aid. Once informed of the programs, 50% of growers expressed interest in participating. Fifty-seven percent of growers manage habitat for other wildlife, although none receive cost-share funding to do so. Participation in cost-share programs could benefit from outreach activities that promote the programs, a reduction of bureaucratic hurdles to participate, and technical support for growers on how to manage habitat for wild bees.

## 1. Introduction

The expansion of modern agriculture has led to the destruction and fragmentation of natural habitat, resulting in a massive loss of biodiversity [1,2]. Beneficial insects, such as bees, are one group of organisms negatively affected by agricultural expansion. These insects provide valuable and, in some cases, critical services to humans, including pest suppression and pollination [3,4]. As natural habitat and beneficial organisms decline, these services will also decline. 

One way to alleviate the loss of biodiversity, and beneficial insects in particular, is through the conservation of non-crop habitats within agricultural landscapes. With ~40% of the Earth’s land area in agricultural production [2], incorporating on-farm habitat conservation practices has the potential to make a substantial contribution to protecting biodiversity. Due to this, governments around the world have developed conservation cost-share programs to encourage farmers to manage non-crop habitat [5]. Previous studies have documented the value of this non-crop habitat in promoting beneficial insects and the services they provide [6,7,8].

In the United States, a number of different conservation cost-share programs exist in which farmers receive financial support to either take cropland out of production and instead plant non-crop habitat (e.g., Conservation Reserve Program, Environmental Quality Incentives Program) or preserve existing natural habitat (e.g., Conservation Stewardship Program) with the goal of preserving biodiversity. In particular, several programs provide specific practices within cost-share programs for farmers to install pollinator habitat [9] (e.g., CP-42 Pollinator Habitat in the Conservation Reserve Program (CRP), 327-Conservation Cover in the Environmental Quality Incentives Program (EQIP); see [10] for a more complete list of available practices). Moreover, as a result of the 2008 USDA Farm Bill, USDA conservationists must now prioritize pollinators and pollinator habitat when determining payments or reviewing and developing conservation practices [11].

These programs could be especially beneficial to farmers growing pollinator-dependent crops. Cranberry is a pollinator-dependent crop [12,13] grown primarily in Wisconsin (USA) and most growers spend thousands of dollars each year on honey bee hive rentals. For example, at 2017 rates of $73 per honey bee hive, stocking densities ranging from 1–18 hives per hectare [14] and average 35 hectares of cranberries per marsh [15], this results in $2555–$45,990 spent on honey bee pollination per year per cranberry farm in Wisconsin. However, native, wild bees are also effective pollinators of cranberry [14,16]. By creating on-farm pollinator habitat, cranberry growers may be able to encourage native bees on their property and harness the economically significant pollination services provided by these bees. Cranberry is the number one fruit crop in Wisconsin contributing nearly $1 billion in value added output to the economy [17]. Furthermore, Wisconsin is the leader in cranberry production producing 60% of the US crop. As such, the actions of Wisconsin cranberry growers have the potential to influence other sectors of the agricultural industry both within Wisconsin (e.g., cherry, apple) and nationally (e.g., cranberry in other growing regions). Wisconsin cranberry growers also often own a significant amount of non-crop land around their cranberry marshes [15], providing plenty of space to install pollinator habitat. Furthermore, their high rate of adoption of integrated pest management (IPM) suggests an appreciation for environmental stewardship.

Despite the possible benefits, participation by Wisconsin cranberry growers in USDA cost-share programs for pollinator conservation was nearly non-existent as of 2011 [18]. Previous studies from different cropping systems identified factors that hinder participation in other conservation programs, including a perceived pest problem from conservation habitat, lack of awareness of the programs, lack of technical knowledge or support to implement the program, and/or a perception that the programs require too much time, space, or money [19,20,21,22]. Theory suggests that in order for farmers to overcome these factors and change their practices, they may also need to experience a shift in their beliefs, values, or attitudes regarding environmental conservation [23,24]. Common factors motivating landowners behavior in relation to wildlife conservation include concern for wildlife populations, desire to protect the land for future generations, and financial incentives [25]. Few studies, however, have investigated the factors that influence adoption of pollinator-specific conservation practices in agroecosystems (but see [23]). By understanding which factors are the most important barriers to, and motivations for, participation, we can more effectively address the challenges and foster adoption of pollinator conservation programs by the growers. 

The objectives of our study were to determine the factors that are most associated with whether Wisconsin cranberry growers (1) actively manage their farms to encourage native bee pollinators, (2) actively manage habitat to protect other wildlife, and (3) participate in on-farm conservation cost-share programs. From this we hoped to determine the factors or variables that motivate or create barriers for Wisconsin cranberry growers to participle in USDA cost-share programs for native bees, and provide practical solutions to increase participation. With recent attention on pollinator conservation at both at the federal and state level, it is important to understand what factors influence whether growers of a pollinator-dependent crop implement management practices with pollinator conservation in mind.

## 2. Materials and Methods 

### 2.1. Grower Survey

We conducted a two-wave mail survey of Wisconsin cranberry growers in June 2011. The survey included 50 questions that sought information on current farming practices, pollination, on-farm conservation, and grower demographics (Supplementary Material, Q1–50). The questions were extensively reviewed and revised based on feedback from cranberry growers, conservation professionals, and extension professors to ensure clarity and completeness. The survey was also reviewed by the University of Wisconsin Survey Center to ensure conformance with best practices in questionnaire design.

Dissemination of the survey was done in partnership with the Wisconsin State Cranberry Growers Association (WSCGA) in order to gain access to the study population while also complying with their privacy policy (i.e., they do not share their mailing list). Two mailings were sent to the entire mailing list (n = 250): (1) an initial mailing with cover letter and survey, and (2) a follow-up postcard reminder one week after the initial mailing [26]. 

### 2.2. Statistical Analysis 

Results were summarized using descriptive statistics, coding analysis, and classification and regression tree (CART) analysis using the package rpart [27] in R (R Core Team, Vienna, Austria [28]). A coding analysis was used to summarize open-ended questions regarding current practices used by growers to conserve wild bees (Q22 and Q25). Responses were first grouped into broad categories (e.g., enhance floral resources) and then within these categories, responses were summarized by management activities (e.g., planting flowers on property). CART analysis was used to determine which factors were most important in predicting the response of growers to specific questions regarding active management of habitat for wild, native bees (Q22); alteration of management to protect wild, native bees (Q25); interest in managing for wild, native bees (Q27); management of habitat for other wildlife (Q29); and interest in participating in a conservation cost-share program to create habitat for wild, native bees (Q39). A CART analysis is a non-parametric method used in order to determine which factors are most important in predicting values of another factor [29,30,31]. For factors with discrete variables (e.g., yes/no responses) a classification tree is used rather than a regression tree. All other survey questions were included as possible predictors in the CART model except for certain questions used as response variables (i.e., for Q22 we excluded Q25 and Q29; for Q25 we excluded Q22 and Q29; for Q29 we excluded Q22). The results of this analysis help identify factors that classify growers into groups with similar responses to a given question. By understanding which factors best predict grower actions, future extension and outreach efforts can be better targeted to address the desired conservation outcomes and the underlying factors influencing growers’ decisions. 

## 3. Results

### 3.1. Survey Response and Demographics

Response was high with 49% of surveys being returned (n = 122/250). Respondents represented a wide range of geographic locations and demographics. Growers responded from 13 counties in Central and Northern Wisconsin—consistent with the cranberry growing regions of Wisconsin (Table 1). 

Ninety-two percent of returned surveys came from the central growing region which accounts for 83% of the total cranberry acreage in Wisconsin (WSCGA). The average response rate by county was slightly higher from counties in the central growing region (45%) as compared to the northern growing region (32%). Notably, 44% of responses were from Wood County, where 30% of all Wisconsin cranberry farms and 26% of cranberry acreage is located in the state [32]. Respondents reported having grown cranberries for an average of 24.5 years (±1.15 SD, median 23, range 4–77). The mean property size was 1115 acres (±159 SD) with cranberry production typically covering 50–149 acres of the total property or an average of 4–13% of the total property. Lastly, 84% of respondents indicated that growing cranberries was their primary source of income.

### 3.2. Active Management for Bees

Although none (0%) of the respondents reported participation in federal cost-share programs for enhancing pollinator habitat, 33% nevertheless actively manage habitat on their farms to encourage native bees on their property without outside assistance from a USDA cost-share program. Of the growers that did habitat enhancements targeted at pollinators, they used two broad approaches: providing floral resources (55%), enhancing nesting resource (36%), or both (6%). In order to provide floral resources growers reported planting or encouraging flowering plants around their marsh (33%) or altering their mowing schedule to leaving flowering weeds on the marsh (21%). Nest site enhancement included building brush piles along the marsh (18%), providing bee boxes (9%), and leaving old mattresses and junk piles nearby for bumble bee nests (9%).

Additionally, 30% of respondents reported that they have altered their crop management in some way to encourage wild bees on their property. This included using reduced-risk pesticides (12%), changing their spray schedule to avoid foraging pollinators (30%), and replacing some pesticide applications with a spring flood (6%) which is a cultural method that can be an effective pest control approach [33].

**Figure 1:**
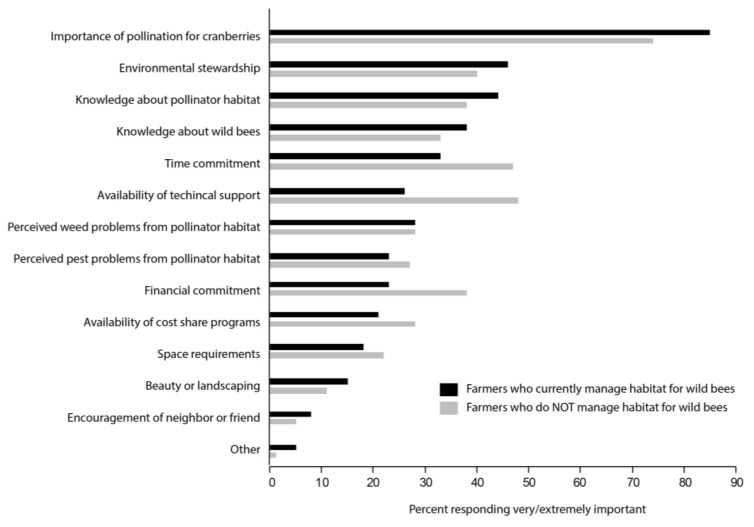
Percent of growers responding “very” or “extremely” to how important each factor is in their decision whether or not to manage for wild bees (Q26).

The three factors that were most often selected as “very or extremely important” in growers’ decision to manage for native bees were (1) the importance of pollination for cranberries, (2) environmental stewardship, and (3) knowledge about pollinator habitat (Figure 1). Of the growers who do not manage for native bees (55%), the most important factors influencing their decision were (1) the importance of pollination for cranberries, (2) the availability of technical support, and (3) time commitment (Figure 1). This may suggests that these growers view pollination as an important management consideration that is best addressed by renting managed honey bees. Renting honey bees is one way that growers can manage for the uncertainty of wild pollinator populations by flooding their farms with managed bees.

The CART analysis indicated that variables associated with grower demographics, management practices, logistics (e.g., time commitment, space requirements), knowledge, past participation in conservation programs, etc., that were collected in this survey could not predict whether growers currently manage habitat for native bees (Q22), have altered their management for wild bees (Q25), or are interested in managing for wild bees in the future (Q27). Regardless of whether or not the growers manage for wild bees, 87% responded that bees are “very or extremely important” to cranberry pollination and 89% reported that they currently rent honey bees for pollination.

### 3.3. Active Management of Wildlife Habitat

**Figure 2:**
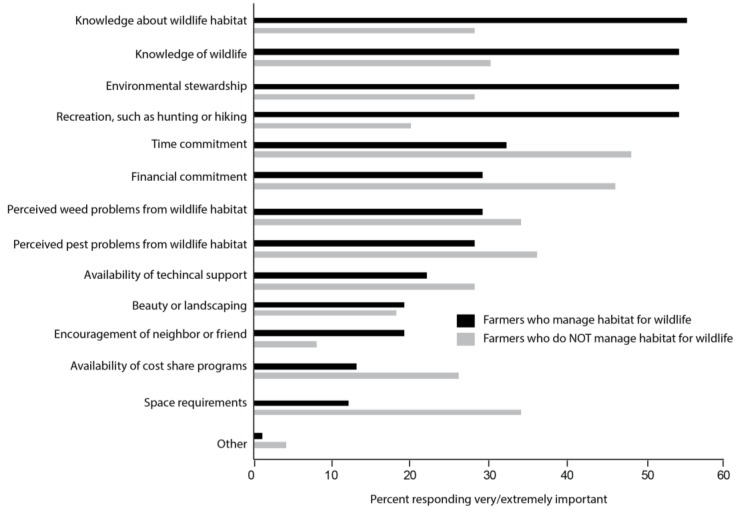
Percent of growers responding “very” or “extremely” to how important each factor is in their decision whether or not to manage habitat for wildlife (Q32).

To understand further how growers perceive on-farm conservation in general, we asked a series of questions regarding management of habitat for wildlife. We found that 57% of growers actively manage habitat for the specific goal of protecting wildlife (e.g., birds, mammals, beneficial insects), although none are receiving cost-share funding to do so. The factors that were most often selected as “very or extremely important” by growers who manage habitat for wildlife habitat were (1) knowledge about wildlife habitat, tied for second were (2) knowledge of wildlife, (2) environmental stewardship, (2) recreation such as hunting or hiking, and (3) time commitment (Figure 2). Of the growers who do not manage for wildlife habitat, the most important factors in their decision making was (1) time commitment, (2) financial commitment, and (3) perceived pest problems from wildlife habitat.

**Figure 3:**
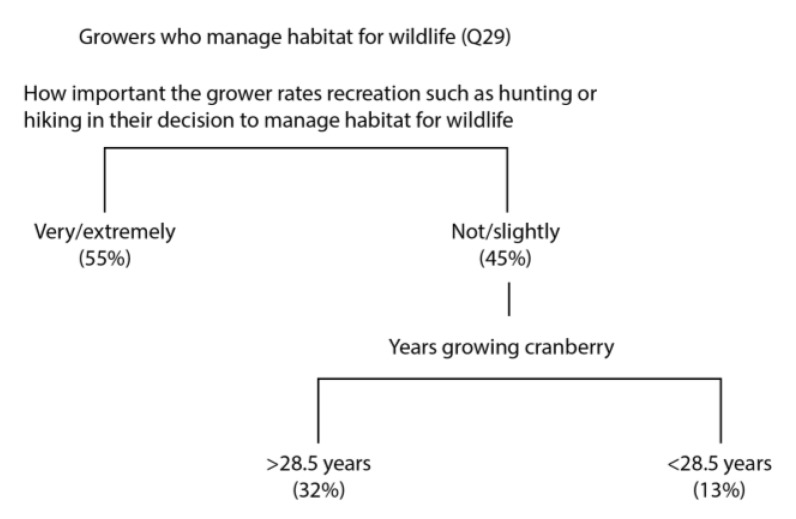
A classification tree that predicts whether growers responded positively to managing habitat for wildlife. Numbers below each tree branch indicate the percent of respondents in that category.

Our results further indicated that the most important predictor variable for whether or not growers manage habitat to protect wildlife was how important they rated recreation (Figure 3); of those who manage habitat, 55% also rated recreation as very/extremely important. For respondents who did not rate recreation as important, the next most important predictor variable was the number of years they had been growing cranberries; 32% of growers who manage habitat for wildlife have been growing cranberries for more than 28.5 years, whereas only 13% have been growing cranberries for less than 28.5 years.

These results suggest that growers who manage for wildlife have knowledge of wildlife and their habitat requirements, a strong environmental stewardship ethic and interest in outdoor recreation such as hunting or hiking. In addition, for farmers who don’t manage habitat for wildlife, perceived time and financial commitment, in addition to possible pest problems, are the most important factors influencing their decision.

### 3.4. Participation in Conservation Cost-Share Programs 

**Figure 4:**
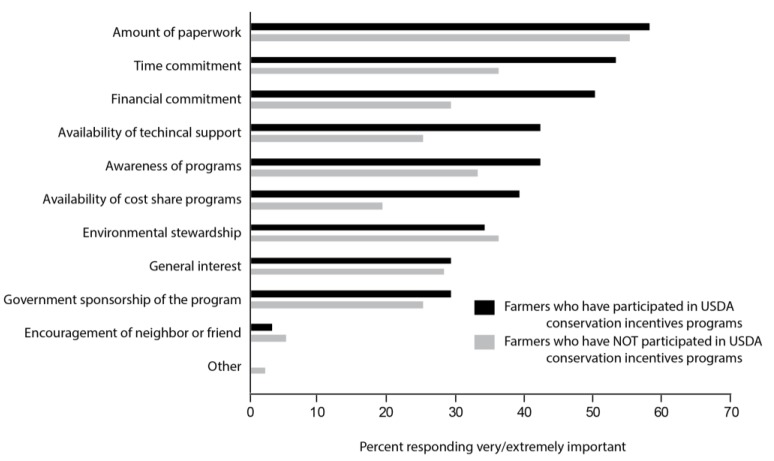
Percent of growers responding “very” or “extremely” to how important each factor is in their decision whether or not to participate in a conservation cost-share program (Q38).

Thirty-one percent of respondents indicated that they currently or previously participated in USDA-sponsored conservation cost-share programs including EQIP (22%), CRP (8%), the Conservation Reserve Enhancement Program (CREP, 2%), and the Wildlife Habitat Incentive Program (WHIP, 2%). None of these programs were targeted at pollinators. However, growers were split on whether they would participate in conservation cost-share programs in the future, and more were unlikely to participate in a USDA-sponsored program (36%) than a non-USDA-sponsored program (26%). We also found that the most important factors for growers who participated in a conservation cost-share program were (1) the amount of paperwork, (2) time commitment, and (3) financial commitment (Figure 4). Of the growers who did not participate in conservation cost-share programs, the most important factors influencing their decision were (1) amount of paperwork, (2) time commitment and environmental stewardship equally, and (3) awareness of the programs.

**Figure 5:**
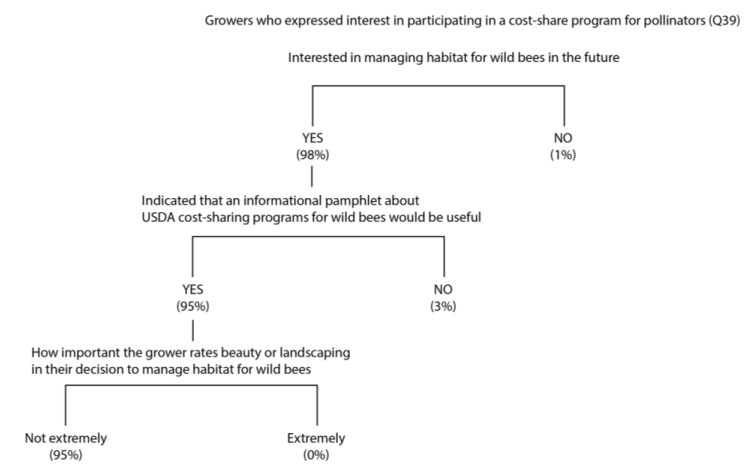
A classification tree that predicts whether growers are interested in participating in a cost-share program for wild bees in the future (Q39). The numbers below each branch indicate the percentage of respondents within each group.

While none of the respondents participate in a cost-share program specifically for pollinator enhancements, only 10% were aware that such programs even exist. When informed of the existence of these programs, 50% of growers expressed interest in participating. The most important predictor variable for whether or not growers were interested in participating in a cost-share program to install pollinator habitat on their property was their interest in managing habitat for bees in the future (Figure 5). The next best predictor variable was whether the grower responded positively to the usefulness of an informational pamphlet about USDA conservation cost-share programs for wild bees. The final predictor variable was how important the respondent rated “beauty” or “landscaping” in their decision process. Of the 2% of respondents who rated beauty and landscaping as “extremely important”, none were interested in managing habitat for bees in the future, possibly because of a perception that this habitat would be unattractive.

### 3.5. Sources of Management Information for Growers

By understanding which sources of information growers use or want to use, more effective extension efforts can target these communication channels. Currently, the most common sources of information are paper newsletters (90%), crop scouts (82%), and the annual meeting of the Wisconsin State Cranberry Growers Association (“Cranberry School”, 81%). Furthermore, 64% of growers indicated that they get information regarding management practices from their neighbors and friends. In the future, growers expressed interest in using email listservs (38%) in addition to paper newsletters (36%), and crop scouts (35%). We also collected information on sources the growers do not want to use in the future with the top three being social networking sites (85%), automatic text message alerts (61%), and email listservs (44%).

## 4. Discussion

Despite their demonstrated commitment to environmental stewardship, participation in pollinator habitat cost-share programs by Wisconsin cranberry growers is low. Through a written survey of Wisconsin cranberry growers, we found that awareness of pollinator habitat cost-share programs is also low. We also found that despite the lack of participation in formal conservation programs, a third of growers are currently managing habitat for pollinators anyway. These same growers were also more likely to manage habitat for other wildlife. Yet, growers who were not managing habitat for bees or other wildlife were deterred by a lack of technical support, and the perceived time and financial commitments required by these cost-share programs. This suggests that outreach and extension efforts could focus both on promoting pollinator habitat cost-share programs as well as providing information and technical support to growers interested in creating pollinator habitat.

One explanation for the low participation rate in pollinator habitat conservation programs is a general dislike or distrust of government cost-share programs. Indeed, several comments on returned surveys suggested that some growers felt it was their duty to care for the environment and, therefore, should not receive government funding to do so. For example, one respondent likened pollinator habitat installation to a business improvement that would increase the sustainability of the business (i.e., the farm) and, therefore, any costs should be incurred by the farm. This could also explain the relatively high proportion (33%) of farmers managing habitat for wild pollinators but not participating in a formal cost-share program. Studies in other farming systems have found a similar pattern where farmers adopt conservation practices without formally participating in government programs [21]. Instead, growers may also be more strongly motivated by their conservation land ethic and appreciation for the outdoors than monetary compensation. Similar to our results, Greiner and Gregg [34] and Banack and Hvengaard [35] found that farmers who implemented conservation practices were most commonly motivated by a moral obligation to care for the environment over economic factors. While financial cost-share programs are one way to motivate growers, appealing to their sense of environmental stewardship may be an equally strong and effective way to increase the conservation of non-crop habitat on farms.

Another explanation for low participation rates is an aversion to bureaucratic hurdles (e.g., time commitment, paperwork). This was also highlighted in the low percentage of growers who expressed interested in participating in federal cost-share programs in the future. Previous research has also found that farmers were discouraged from participating in conservation practices on their farms because of the complexity of paperwork involved [20,21,22].

Finally, the lack of awareness of the available programs for pollinator conservation certainly hinders the participation of growers. This is consistent with previous work in the apple system in New York and Pennsylvania where only 9–25% of growers were aware that the programs existed [36] although growers demonstrated a clear interest in wild pollinators. In Michigan, where adoption of the USDA pollinator habitat programs has been high, the key to success has been active promotion of the programs (e.g., high engagement of FSA/NRCS personnel with the farm community), as well as good financial incentive of up to a 90% cost-share [37]. In other regions of the country and the world, successful implementation of conservation programs has partially been a result of engaged conservation professionals developing personal relationships with the farmers and promoting the benefits of conservation through workshops and one-on-one assistance [19,22,38]. In the United States, Farm Bill conservation program implementation is controlled at the state level by the local NRCS offices which have flexibility on which programs they promote and how they promote the programs [39]. This implementation strategy could explain the wide variability in participation rates in different regions of the country.

This study highlights four key areas that can be addressed to increase participation in cost-share programs aimed at creating pollinator habitat. First, the specific programs and practices intended for pollinator conservation need promoting. Only a small percentage of Wisconsin cranberry growers (10%) were aware of the programs, although half of the growers were interested in these programs once they were made aware. Since 80–90% of the cranberry growers reported getting their management information from industry newsletters, crop scouts, and Cranberry School, these would be appropriate outlets through which to promote conservation programs. Survey respondents also listed “email listservs” as both the preferred and non-preferred sources of information, demonstrating the importance of disseminating information through a diversity of channels. 

Second, the paperwork required to participate in these programs needs to be reduced. Lowering the bureaucratic hurdles to participation would greatly reduce the time commitment and make participation easier. In order to participate in USDA-sponsored conservation cost-share programs, growers have to go through a long process of determining eligibility through the Farm Service Agency (FSA), developing conservation plans, selecting appropriate programs and practices, and submitting proposals to the FSA through the USDA Natural Resources Conservation Service (NRCS) [40]. In addition to all of the paperwork already required by the growers regarding application of pesticides, water use, and permits for marsh renovation and expansion, extra paperwork may discourage growers from participating in cost-share programs [22]. Streamlining the process by reducing the amount of paperwork could greatly increase participation.

Third, providing accessible technical support could provide the knowledge required to establish and maintain the plantings. In this population of growers, technical hurdles were seen as important and growers expressed the importance of knowledge of wildlife habitat and requirements. Engaged, on-the-ground technical support could assist with the design and implementation of conservation projects that include pollinator habitat [41,42]. However, without a person available to direct the growers, answer their questions, and provide practical on-the-ground solutions, few growers will attempt to install pollinator habitat. Several informational pamphlets exist to help growers plan their pollinator planting [9,43,44], but with many farmers still lacking high speed internet [45] accessing these resources can be difficult.

Lastly, program administrators could establish a peer-mentoring program to connect growers who are currently managing pollinator habitat with growers who are interested in managing pollinator habitat. Sixty-four percent of growers indicated that they get information regarding management practices from their neighbors and friends, suggesting that peer mentoring could be an effective way to increase participation. Providing growers with an example of an established pollinator habitat planting, or demonstration site, and connecting them with a grower who has gone through the process has the potential to increase the success of interested growers while reducing the demand put on agency personnel to address every concern from new participants. Demonstration sites can be an effective tool for conservation science in order to demonstrate the effectiveness and feasibility of implementing conservation management practices in a real-world setting [46]. Although it is beyond the scope of this research project, further thought and development will need to be given to create incentives for growers acting as mentors to be part of the program.

The results and conclusions of this study are likely applicable to other sectors of the agricultural industry outside of the Wisconsin cranberry grower community. The commitment to environmental stewardship is a common thread across agricultural systems in both Wisconsin (e.g., Wisconsin Healthy Grown Potatoes [47]) and elsewhere (e.g., Eco-Apple [48] www.redtomato.org/eco-apple/). The lack of engagement in government-sponsored conservation programs is possibly a reflection of a broader politically conservative philosophy common throughout rural America [49]. Therefore, we would expect other growers to have similar attitudes and behaviors to Wisconsin cranberry growers.

## 5. Conclusions

Conserving non-crop habitats within the agricultural landscape is one approach to protecting and enhancing biodiversity worldwide. Governmental cost-share programs provide a way for farmers to implement habitat establishment on their farms. The recommendations described above have the potential to greatly increase participation in cost-share programs for pollinator habitat by Wisconsin cranberry growers, as well as growers of other crops, by highlighting some of the barriers to participation. Increasing participation among fruit growers, who depend on pollinators to produce a crop [50], would not only benefit the native pollinator communities on their farms [8], it would also enhance other beneficial insects and the ecosystem services they provide [51]. By altering the agricultural landscape by implementing on-farm conservation habitat, farmers can turn a resource-poor landscape into one that supports biodiversity [1].

Since conducting this survey six years ago, pollinator protection has become an area of national (and international) focus. In 2015, then-US president Barack Obama issued a Presidential Memorandum initiating the National Strategy to Promote Pollinator Health [52]. This action lead to the promotion of pollinator protection on all federal properties and has been followed-up by individual state pollinator protection plans. The recommended actions described above, along with the momentum at the state and federal level, have the potential to lead to a significant increase in the creation and protection of pollinator habitat in the agricultural landscape.

## Figures and Tables

**Table 1:** Summary of farm numbers and number of responding farms by county including the total number of cranberry farms per county, the number of surveys returned from each county, the percentage of cranberry farms found in each county, the percentage of respondents from each county (number of respondents per county/total surveys returned), and the response rate by county (surveys returned/number of farms).

Growing Region	County	Number of Farms (2012 NASS)	Number of Farms Responding (2011)	Percent of Total Cranberry Farms in Wisconsin (%)	Percent of Respondents (%) *	Response Rate (%) **
Central	Adams	9	7	4	6	78
	Buffalo	1	0	<1	0	0
	Jackson	36	23	15	19	64
	Juneau	8	11	3	9	>100
	Monroe	62	22	26	18	35
	Portage	14	3	6	2	21
	Waushara	1	0	<1	0	0
	Wood	72	46	30	38	64
	**SUB-TOTAL**	**203**	**112**	**84**	**92**	**55**
Northern	Ashland	1	0	<1	0	0
	Burnett	1	0	<1	0	0
	Iron	3	0	1	0	0
	Lincoln	1	2	0	2	>100
	Oconto	2	0	1	0	0
	Oneida	11	5	5	4	45
	Price	3	2	1	2	67
	Rusk	1	0	<1	0	0
	Sawyer	8	1	3	1	13
	Vilas	5	4	2	3	80
	Washburn	2	1	1	1	50
	**SUB-TOTAL**	**38**	**15**	**16**	**12**	**39**
	**TOTAL**	**241**	**127**			

* Sum may not equal 100% because some respondents reported growing cranberries in multiple counties and are, therefore, tallied twice in this table. ** May be >100% because the data on number of farms by county was collected in 2012 by USDA NASS and the survey data was collected in 2011.

## Supplementary Materials

The following is available online at http://www.mdpi.com/2075-4450/8/3/79/s1: A complete list of the survey questions mailed to the membership of the Wisconsin Cranberry Growers Association: Cranberry Grower Survey.
