# Supplementary material for: Understanding Barriers to Participation in Cost-Share Programs For Pollinator Conservation by Wisconsin (USA) Cranberry Growers
Source: Insects. 2017 Aug 1;8(3):79. doi: 10.3390/insects8030079 (PMC5620699; doi:10.3390/insects8030079)
Supplement: Supplementary file 1 [file insects-08-00079-s001.pdf]

Appendix A: Survey sent to Wisconsin cranberry growers.

THE UNIVERSITY of WISCONSIN MADISON

Cranberry Grower Survey

This study is being conducted in order to understand how cranberry growers regard on-farm conservation programs in general and pollinator conservation programs in particular.

Your answers will be kept confidential, and not released in a way that would allow you to be identified.

1. Are you currently acting as the primary farm operator for your cranberry farm? Acting as the primary farm operator would include handling day to day operations and making decisions regarding farming practices for cranberry production done on your farm.

- ◯ Yes → **Go to question 2**
- ◯ No

We would appreciate your assistance in passing this survey on to the person who is the primary farm operator for your Cranberry farm.

If you are unable to pass the survey on to that person, please make note of that here and return the blank survey in the enclosed postage paid envelope.

Thank you!

MANAGEMENT OF CRANBERRY MARSHES

This section is about current management practices and where you obtain information on management practices.

2. Within the past 5 years, if you ever irrigated, in general, what time of day did you irrigate your cranberry beds? (*Check all that apply*)

- ☐ Before 8:00 a.m.
- ☐ Between 8:00 a.m. and 3:00 p.m.
- ☐ Between 3:00 p.m. and 7:00 p.m.
- ☐ After 7:00 p.m.
- ☐ I never irrigated over the past 5 years

3. Within the past 5 years, if you applied any of the following substances, what time of day did you usually apply them to your cranberry beds? (*Check all that apply*)

	Before 8:00 a.m.	Between 8:00 a.m. and 3:00 p.m.	Between 3:00 p.m. and 7:00 p.m.	After 7:00 p.m.	I did not apply in past 5 years
**a. Insecticide**	☐	☐	☐	☐	☐
**b. Herbicide**	☐	☐	☐	☐	☐
**c. Fungicide**	☐	☐	☐	☐	☐

4. Within the past 5 years, if you applied any of the following substances, how many applications, on average, did you spray per year of each of the following? If you did not spray at all, please write '0' for that substance.

	Number of applications per year
**a. Insecticide**	☐
**b. Herbicide**	☐
**c. Fungicide**	☐
**d. Other, please describe** ________	☐

5. Within the past 5 years, did you ever use the following types of insecticide products on your cranberry beds?

	Yes	No
**a. Insect growth regulators such as Confirm or Intrepid**	○	○
**b. Neonicotinoids such as Assail**	○	○
**c. Organophosphates such as Diazinon or Lorsban**	○	○
**d. Some other products, please describe** ________	○	○

6. Is your farm certified organic?

○ Yes
○ No

7. Do you hire a scout to monitor for insect pests?

○ Yes
○ No

8. Within the past 5 years, which of the following methods did you use to determine when to spray for insect pests? Did you use…

	Yes	No
**a. ...a calendar schedule?**	○	○
**b. ...scout reports?**	○	○
**c. ...some other method? please describe** ________	○	○

9. Within the past 5 years, what insect pest management strategies did you use in addition to or instead of insecticides? Did you use…

	Yes	No
**a. ...flooding?**	○	○
**b. ...biological control?**	○	○
**c. ...sanding?**	○	○
**d. ...some other strategy? please describe** ________	○	○

10. Below is a list of sources of information about pest, nutrient, water, frost and other management practices. For each source of information, please indicate if you have used that source in the past, if you are using it now, if you want to use it in the future, or if you have NEVER used that source of information, and would not want to in the future. (*Check all that apply*)

	Used it in the past	Use it now	Want to use it in the future	Never used it and will not in the future
a. Crop Scout	☐	☐	☐	☐
b. University Extension Agent	☐	☐	☐	☐
c. Natural Resources Conservation Service (NRCS) Staff	☐	☐	☐	☐
d. Neighbor or friend	☐	☐	☐	☐
e. Cranberry School	☐	☐	☐	☐
f. Information hotline (phone)	☐	☐	☐	☐
g. Newsletter (paper)	☐	☐	☐	☐
h. Email listserv	☐	☐	☐	☐
i. Text message (automatic alerts)	☐	☐	☐	☐
j. Social networking site (Facebook, Twitter)	☐	☐	☐	☐
k. Website (please list below) ☐______	☐	☐	☐	☐
l. Other (please list below) ☐______	☐	☐	☐	☐

POLLINATORS - WILD AND MANAGED

This section is about your current pollination practices and your awareness about wild bees and pollinator habitat.

11. Do you currently rent honey bees for pollination?

- ○ Yes
- ○ No → **Go to question 13**

12. How many hives per acre?

☐ hives

13. Have you ever rented honey bees for pollination in the past?

- ○ Yes
- ○ No

14. Do you use commercial bumble bees for pollination?

- ○ Yes
- ○ No → **Go to question 16**

15. How many hives per acre?

☐ hives

16. Have you noticed a change in the *availability* of honey bee hives in the past 5 years?

- ○ Yes → **Please explain** ☐
- ○ No

17. Have you observed a change in the *quality* of rented honey bee hives in the past 5 years?

- ○ Yes → **Please explain** ☐
- ○ No

18. How familiar are you with Colony Collapse Disorder (CCD)?

- ○ Not at all familiar
- ○ Slightly familliar
- ○ Somewhat familiar
- ○ Very familiar
- ○ Extremely familiar

19. Can you distinguish between wild bees and honey bees?

- ○ Yes
- ○ No

20. How important do you think honey bees are for cranberry pollination?

- ○ Not at all important
- ○ Slightly important
- ○ Somewhat important
- ○ Very important
- ○ Extremely important

21. Are you aware of any wild bees on your property?

- ○ Yes
- ○ No

22. **Do you actively manage *habitat* to encourage wild bees on your property?**

○ Yes → **Please explain** [ ]

○ No → **Go to question 24**

23. **If you do manage habitat for wild bees, did you receive cost-share funding to do this?**

○ Yes → **Through whom?** [ ]

○ No

24. **Are you aware of cost-share programs for establishing pollinator habitat?**

○ Yes

○ No

25. **Have you altered your management *in any way* to encourage wild bees on your property?**

○ Yes → **Please explain** [ ]

○ No

26. **How important are each of the following in your decision about whether or not to manage for wild bees?**

	Not at all	Slightly	Somewhat	Very	Extremely	Not applicable
a. Financial commitment	○	○	○	○	○	○
b. Time commitment	○	○	○	○	○	○
c. Space requirements	○	○	○	○	○	○
d. Availability of cost share programs	○	○	○	○	○	○
e. Availability of technical support	○	○	○	○	○	○
f. Environmental stewardship	○	○	○	○	○	○
g. Encouragement of neighbor or friend	○	○	○	○	○	○
h. Knowledge about wild bees	○	○	○	○	○	○
i. Knowledge about pollinator habitat	○	○	○	○	○	○
j. Perceived weed problems from pollinator habitat	○	○	○	○	○	○
k. Perceived pest problems from pollinator habitat	○	○	○	○	○	○
l. Beauty or landscaping	○	○	○	○	○	○
m. Importance of pollination for cranberries	○	○	○	○	○	○
n. Other (please explain) [ ]	○	○	○	○	○	○

27. If you do not currently manage for wild bees on your property would you be interested in managing for wild bees in the future?

○ Yes

○ No

28. Which of the following would be useful to you?

	Useful	Not useful
**a. Field day demonstration of a pollinator habitat project**	○	○
**b. Informational pamphlet about wild bees**	○	○
**c. Field guide to wild bees**	○	○
**d. Informational pamphlet about USDA cost-sharing programs for wild bees**	○	○
**e. Website about wild bees**	○	○
**f. Website about pollinator habitat**	○	○
**g. Website about USDA cost-sharing programs for wild bees**	○	○
**h. Other (please explain)** ________	○	○

ON-FARM CONSERVATION PROGRAMS

The next questions are about on-farm conservation programs in general.

29. Do you currently manage habitat (prairie plantings, woodlots, wetland, etc.) for the specific goal of protecting wildlife (birds, beneficial insects, mammals, etc.)?

○ Yes

○ No → **Go to question 32**

30. If yes, do you receive cost-share funding to support these conservation management activities?

○ Yes

○ No → **Go to question 32**

31. Through whom do you receive cost-share funding to support these conservation management activities?

____

32. How <u>important</u> are the following in your decision whether or not to manage habitat for <u>wildlife</u> such as birds, beneficial insects, and mammals?

	Not at all	Slightly	Somewhat	Very	Extremely	Not applicable
a. Financial commitment	○	○	○	○	○	○
b. Time commitment	○	○	○	○	○	○
c. Space requirements	○	○	○	○	○	○
d. Availability of cost share programs	○	○	○	○	○	○
e. Availability of technical support	○	○	○	○	○	○
f. Environmental stewardship	○	○	○	○	○	○
g. Encouragement of neighbor or friend	○	○	○	○	○	○
h. Knowledge of wildlife	○	○	○	○	○	○
i. Knowledge of wildlife habitat	○	○	○	○	○	○
j. Perceived weed problems from wildlife habitat	○	○	○	○	○	○
k. Perceived pest problems from wildlife habitat	○	○	○	○	○	○
l. Beauty or landscaping	○	○	○	○	○	○
m. Recreation, such as hunting or hiking	○	○	○	○	○	○
n. Other (please explain) ________	○	○	○	○	○	○

33. Do you or have you ever participated in any of the following United States Department of Agriculture (USDA)-sponsored conservation incentive programs? (*Check all that apply*.)

- ☐ Conservation Reserve Program (CRP)
- ☐ Conservation Reserve Enhancement Program (CREP)
- ☐ Environmental Quality Incentives Program (EQIP)
- ☐ Wildlife Habitat Incentives Program (WHIP)
- ☐ Other, please explain ________

34. Do you currently participate in the Whole Farm Planning Incentives Program (WFPIP)?

- ○ Yes
- ○ No

35. Do you currently participate in any programs that address the following environmental/conservation issues? (*Check all that apply*.)

- ☐ Nutrient management
- ☐ Pest management
- ☐ Water quality and conservation
- ☐ Soil conservation

36. In the future, how likely are you to participate in a <u>USDA-sponsored</u> conservation incentive program?

- ◯ Extremely unlikely
- ◯ Very unlikely
- ◯ Somewhat unlikely
- ◯ Not sure
- ◯ Somewhat likely
- ◯ Very likely
- ◯ Extremely likely

37. In the future, how likely are you to participate in a <u>non-USDA-sponsored</u> conservation incentive program?

- ◯ Extremely unlikely
- ◯ Very unlikely
- ◯ Somewhat unlikely
- ◯ Not sure
- ◯ Somewhat likely
- ◯ Very likely
- ◯ Extremely likely

38. **How important are the following in your decision whether or not to participate in a conservation incentive program?**

	Not at all	Slightly	Somewhat	Very	Extremely	Not applicable
a. Financial commitment	○	○	○	○	○	○
b. Time commitment	○	○	○	○	○	○
c. Availability of cost share programs	○	○	○	○	○	○
d. Availability of technical support	○	○	○	○	○	○
e. Environmental stewardship	○	○	○	○	○	○
f. Encouragement of neighbor or friend	○	○	○	○	○	○
g. Awareness of programs	○	○	○	○	○	○
h. General interest	○	○	○	○	○	○
i. Amount of paperwork	○	○	○	○	○	○
j. Government sponsorship of program	○	○	○	○	○	○
k. Other (please explain) ____________	○	○	○	○	○	○

39. **Would you be interested in participating in a cost-share conservation program to enhance wild bees through the planting of pollinator habitat on your property?**

○ Yes
○ No

40. **Do you know who your NRCS officer or representative is?**

○ Yes
○ No

41. **Are you comfortable working with or getting information from your NRCS officer or representative?**

○ Yes
○ No

YOUR BACKGROUND

These last questions are about you.

42. What is your age?

- ◯ Less than 25 years old
- ◯ 25-34
- ◯ 35-44
- ◯ 45-54
- ◯ 55-64
- ◯ 65 or older

43. What is your gender?

- ◯ Male
- ◯ Female

44. In which county (or counties) do you grow cranberries?

[ ]

45. How many acres of cranberries do you manage?

- ◯ Less than 50 acres
- ◯ 50 – 149 acres
- ◯ 150 – 300 acres
- ◯ Over 300 acres

46. How many total acres do you own or manage, including all cranberry and non-cranberry?

[ ] acres

47. How long have YOU been growing cranberries?

[ ] years

48. How long has YOUR FAMILY been growing cranberries?

[ ] years

49. Do you regularly attend Cranberry School?

- ◯ Yes
- ◯ No

50. Is growing cranberries your primary source of income?

- ◯ Yes
- ◯ No

THANK YOU for your participation!

If you have any questions or concerns, please contact Hannah Gaines by phone (774-392-0498) or email (hgaines@gmail.com).

If there is anything else you would like us to know, please use the space below to do so.
